# 4-{[4-(3,5-Dimeth­oxy­benzamido)­phen­yl]sulfan­yl}-*N*-methyl­pyridine-2-carboxamide

**DOI:** 10.1107/S1600536811006490

**Published:** 2011-03-12

**Authors:** Ting-Ting Huang, Na-Na Meng, Xiao-Yu Qing, Luo-Ting Yu

**Affiliations:** a State Key Laboratory of Biotherapy and Cancer Center, West China Hospital, West China Medical School, Sichuan University, Chengdu 610041, People’s Republic of China

## Abstract

There are two independent mol­ecules in the asymmetric unit of the title compound, C_22_H_21_N_3_O_4_S. The central benzene ring makes dihedral angles of 74.28 (6) and 68.84 (6)° with the pyridine and 3,5-dimeth­oxy­phenyl rings, respectively, in one molecule [86.66 (6) and 81.14 (6)° respectively, in the other]. Each of the mol­ecules forms a centrosymmetric dimer with another mol­ecule *via* pairs of inter­molecular N—H⋯O hydrogen bonds. These hydrogen bonds connect the N—H groups and the O atoms of the carbonyl groups next to the 3,5-dimeth­oxy­phenyl rings. Additional inter­molecular N—H⋯O inter­actions link the dimers in the crystal structure.

## Related literature

For related compounds and their biological activity, see: Khire *et al.* (2004 ▶); Dominguez *et al.* (2007 ▶).
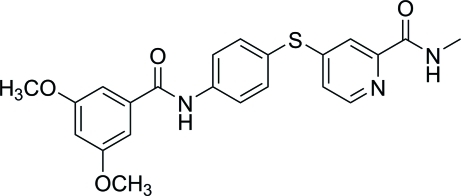


## Experimental

### 

#### Crystal data

C_22_H_21_N_3_O_4_S
*M* *_r_* = 423.49
Triclinic, 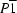

*a* = 11.1165 (5) Å
*b* = 13.5678 (6) Å
*c* = 15.3183 (6) Å
α = 102.723 (4)°
β = 105.949 (4)°
γ = 90.975 (4)°
*V* = 2159.51 (16) Å^3^
*Z* = 4
Mo *K*α radiation
μ = 0.18 mm^−1^
*T* = 293 K
0.24 × 0.20 × 0.17 mm

#### Data collection

Oxford Diffraction Xcalibur Eos diffractometer
Absorption correction: multi-scan (*CrysAlis PRO*; Oxford Diffraction, 2010 ▶) *T*_min_ = 0.989, *T*_max_ = 1.0
18448 measured reflections
8806 independent reflections
4117 reflections with *I* > 2σ(*I*)
*R*_int_ = 0.032

#### Refinement

*R*[*F* ^2^ > 2σ(*F* ^2^)] = 0.044
*wR*(*F* ^2^) = 0.065
*S* = 1.00
8806 reflections
547 parameters
H-atom parameters constrained
Δρ_max_ = 0.24 e Å^−3^
Δρ_min_ = −0.23 e Å^−3^

### 

Data collection: *CrysAlis PRO* (Oxford Diffraction, 2010 ▶); cell refinement: *CrysAlis PRO*; data reduction: *CrysAlis PRO*; program(s) used to solve structure: *SHELXS97* (Sheldrick, 2008 ▶); program(s) used to refine structure: *SHELXL97* (Sheldrick, 2008 ▶); molecular graphics: *OLEX2* (Dolomanov *et al.*, 2009 ▶); software used to prepare material for publication: *OLEX2*.

## Supplementary Material

Crystal structure: contains datablocks I, global. DOI: 10.1107/S1600536811006490/im2258sup1.cif

Structure factors: contains datablocks I. DOI: 10.1107/S1600536811006490/im2258Isup2.hkl

Additional supplementary materials:  crystallographic information; 3D view; checkCIF report

## Figures and Tables

**Table 1:** Hydrogen-bond geometry (Å, °)

*D*—H⋯*A*	*D*—H	H⋯*A*	*D*⋯*A*	*D*—H⋯*A*
N1—H1⋯O4^i^	0.86	2.12	2.941 (2)	159
N3—H3⋯O7^ii^	0.86	2.22	2.922 (2)	138
N4—H4⋯O8^iii^	0.86	2.15	2.985 (2)	163
N6—H6⋯O3^iv^	0.86	2.30	2.957 (2)	133

Symmetry codes: (i) 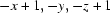; (ii) 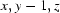; (iii) 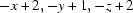; (iv) 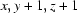.

**Fig. 1.**
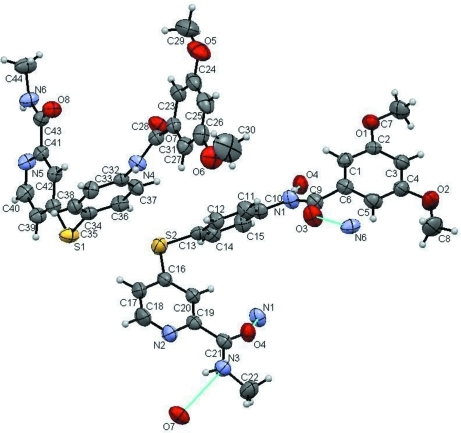
Molecular structure of the title compound with displacement ellipsoids drawn at the 30% probability level.

**Fig. 2.**
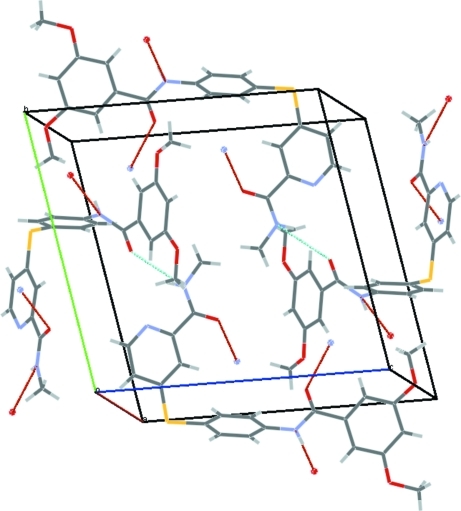
Packing diagram of the title compound.

## supplementary crystallographic information

### Comment

Sorafenib is of great importance owing to its antitumor properties (Khire *et al.*, 2004; Dominguez *et al.*, 2007). The title compound, as one of its derivatives, possessed even better *in vitro* anticancer activity against both two tumor cell lines (HCT116 and HEPG2). As a potent antitumor drug, we report here its crystal structure. In the title molecule, C_22_H_21_N_3_O_4_S, (Fig. 1), the phenyl ring makes dihedral angles of 78.54 (6)° and 75.30 (6)° with the pyridine ring and the 3,5-dimethoxyphenyl ring, respectively. In crystal, the molecules form centrosymmetric dimers *via* a pair of intermolecular N–H···O hydrogen bonds. In the crystal structure, intermolecular N—H···O hydrogen-bonding interactions between the dimers form an infinite three-dimensional structure (Table 1 and Fig. 2).

### Experimental

To the suspension of anhydrous potassium carbonate (0.69 g, 5.00 mmol) and 4-(4-aminophenylthio)-*N*-methylpicolinamide (0.52 g, 2.00 mmol) in 7.00 ml THF was added dropwise 3,5-dimethoxybenzoyl chloride (0.42 g, 2.10 mmol). After being stirred at room temperature for 2 h, the mixture was extracted with 30 ml e thyl acetate and 30 ml brine for three times and the combined organic layers were dried over anhydrous sodium sulfate. Then the solution was concentrated under vacuum, and the residue was recrystallized from ethanol to give the title compound, with 32.13% yield. Crystals suitable for X-ray analysis were obtained by slow evaporation from an ethanolic solution at room temperature.

### Refinement

H atoms at N1 and N3 were located in a difference map and refined isotropically. The remaining H atoms were positioned geometrically (C—H = 0.93–0.96 Å) and refined using a riding model, with *U*_iso_(H) = 1.2–1.5 *U*_eq_(C).

### Crystal data

**Table:**

C_22_H_21_N_3_O_4_S	*Z* = 4
*M**_r_* = 423.49	*F*(000) = 888
Triclinic, *P*1	*D*_x_ = 1.303 Mg m^−^^3^
Hall symbol: -P 1	Mo *K*α radiation, λ = 0.7107 Å
*a* = 11.1165 (5) Å	Cell parameters from 5046 reflections
*b* = 13.5678 (6) Å	θ = 2.9–29.2°
*c* = 15.3183 (6) Å	µ = 0.18 mm^−^^1^
α = 102.723 (4)°	*T* = 293 K
β = 105.949 (4)°	Block, orange
γ = 90.975 (4)°	0.24 × 0.20 × 0.17 mm
*V* = 2159.51 (16) Å^3^	

### Data collection

**Table:**

Oxford Diffraction Xcalibur Eos diffractometer	8806 independent reflections
Radiation source: fine-focus sealed tube	4117 reflections with *I* > 2σ(*I*)
graphite	*R*_int_ = 0.032
Detector resolution: 16.0874 pixels mm^-1^	θ_max_ = 26.4°, θ_min_ = 2.9°
ω scans	*h* = −13→13
Absorption correction: multi-scan (*CrysAlis PRO*; Oxford Diffraction, 2010)	*k* = −16→16
*T*_min_ = 0.989, *T*_max_ = 1.0	*l* = −19→19
18448 measured reflections	

### Refinement

**Table:**

Refinement on *F*^2^	Primary atom site location: structure-invariant direct methods
Least-squares matrix: full	Secondary atom site location: difference Fourier map
*R*[*F*^2^ > 2σ(*F*^2^)] = 0.044	Hydrogen site location: inferred from neighbouring sites
*wR*(*F*^2^) = 0.065	H-atom parameters constrained
*S* = 1.00	*w* = 1/[σ^2^(*F*_o_^2^) + (0.0126*P*)^2^] where *P* = (*F*_o_^2^ + 2*F*_c_^2^)/3
8806 reflections	(Δ/σ)_max_ = 0.001
547 parameters	Δρ_max_ = 0.24 e Å^−^^3^
0 restraints	Δρ_min_ = −0.23 e Å^−^^3^

### Special details

**Table:**

Experimental. CrysAlisPro, Oxford Diffraction Ltd., Version 1.171.33.66 (release 28-04-2010 CrysAlis171 .NET) (compiled Apr 28 2010,14:27:37) Empirical absorption correction using spherical harmonics, implemented in SCALE3 ABSPACK scaling algorithm.
Geometry. All e.s.d.'s (except the e.s.d. in the dihedral angle between two l.s. planes) are estimated using the full covariance matrix. The cell e.s.d.'s are taken into account individually in the estimation of e.s.d.'s in distances, angles and torsion angles; correlations between e.s.d.'s in cell parameters are only used when they are defined by crystal symmetry. An approximate (isotropic) treatment of cell e.s.d.'s is used for estimating e.s.d.'s involving l.s. planes.
Refinement. Refinement of *F*^2^ against ALL reflections. The weighted *R*-factor *wR* and goodness of fit *S* are based on *F*^2^, conventional *R*-factors *R* are based on *F*, with *F* set to zero for negative *F*^2^. The threshold expression of *F*^2^ > σ(*F*^2^) is used only for calculating *R*-factors(gt) *etc*. and is not relevant to the choice of reflections for refinement. *R*-factors based on *F*^2^ are statistically about twice as large as those based on *F*, and *R*- factors based on ALL data will be even larger.

### Fractional atomic coordinates and isotropic or equivalent isotropic displacement parameters (Å^2^)

**Table:**

	*x*	*y*	*z*	*U*_iso_*/*U*_eq_	
S1	0.65660 (6)	0.37812 (4)	1.12524 (4)	0.05655 (19)	
O1	0.39178 (13)	0.32537 (11)	0.17737 (9)	0.0536 (4)	
O2	0.33947 (17)	−0.01920 (13)	0.01326 (11)	0.0862 (6)	
O3	0.64974 (15)	0.00594 (12)	0.34157 (10)	0.0708 (5)	
O4	0.64438 (14)	−0.26093 (10)	0.57675 (10)	0.0568 (4)	
N1	0.56894 (16)	0.13586 (12)	0.42239 (12)	0.0541 (5)	
H1	0.5175	0.1823	0.4170	0.065*	
N2	0.89197 (18)	−0.23172 (14)	0.78726 (14)	0.0582 (5)	
N3	0.74299 (16)	−0.37746 (14)	0.64935 (12)	0.0553 (5)	
H3	0.8025	−0.3878	0.6951	0.066*	
C1	0.48731 (19)	0.20452 (16)	0.25279 (15)	0.0453 (6)	
H1A	0.5205	0.2565	0.3056	0.054*	
C2	0.4130 (2)	0.22594 (17)	0.17137 (16)	0.0409 (6)	
C3	0.3640 (2)	0.14940 (17)	0.09286 (15)	0.0513 (6)	
H3A	0.3123	0.1638	0.0389	0.062*	
C4	0.3926 (2)	0.05029 (18)	0.09486 (17)	0.0553 (7)	
C5	0.4657 (2)	0.02726 (17)	0.17531 (17)	0.0555 (7)	
H5	0.4841	−0.0392	0.1764	0.067*	
C6	0.5116 (2)	0.10566 (18)	0.25486 (16)	0.0460 (6)	
C7	0.3120 (2)	0.35033 (16)	0.09594 (15)	0.0696 (7)	
H7B	0.3508	0.3355	0.0462	0.104*	
H7C	0.2990	0.4212	0.1096	0.104*	
H7A	0.2327	0.3110	0.0773	0.104*	
C8	0.3678 (2)	−0.12126 (17)	0.00739 (16)	0.0935 (10)	
H8C	0.4572	−0.1244	0.0242	0.140*	
H8B	0.3313	−0.1602	−0.0553	0.140*	
H8A	0.3342	−0.1485	0.0493	0.140*	
C9	0.5843 (2)	0.07740 (18)	0.34252 (16)	0.0508 (6)	
C10	0.6310 (2)	0.12627 (15)	0.51350 (16)	0.0461 (6)	
C11	0.7543 (2)	0.10139 (15)	0.53712 (16)	0.0557 (7)	
H11	0.7976	0.0900	0.4923	0.067*	
C12	0.8126 (2)	0.09347 (15)	0.62633 (16)	0.0520 (6)	
H12	0.8951	0.0761	0.6413	0.062*	
C13	0.7502 (2)	0.11111 (15)	0.69480 (15)	0.0465 (6)	
C14	0.6284 (2)	0.13777 (15)	0.67162 (16)	0.0544 (6)	
H14	0.5857	0.1508	0.7168	0.065*	
C15	0.5693 (2)	0.14532 (15)	0.58157 (16)	0.0551 (6)	
H15	0.4871	0.1634	0.5667	0.066*	
C16	0.8519 (2)	−0.02610 (16)	0.79660 (15)	0.0460 (6)	
C17	0.9419 (2)	−0.05579 (18)	0.86601 (15)	0.0579 (7)	
H17	0.9908	−0.0079	0.9174	0.069*	
C18	0.9577 (2)	−0.1577 (2)	0.85779 (17)	0.0688 (8)	
H18	1.0190	−0.1761	0.9051	0.083*	
C19	0.8052 (2)	−0.20169 (17)	0.72111 (15)	0.0430 (6)	
C20	0.78341 (19)	−0.10110 (16)	0.72188 (14)	0.0431 (6)	
H20	0.7235	−0.0845	0.6728	0.052*	
C21	0.7239 (2)	−0.28288 (18)	0.64210 (16)	0.0450 (6)	
C22	0.6663 (2)	−0.46444 (16)	0.58221 (16)	0.0773 (8)	
H22B	0.5870	−0.4711	0.5949	0.116*	
H22C	0.7088	−0.5248	0.5874	0.116*	
H22A	0.6526	−0.4546	0.5201	0.116*	
S2	0.83017 (6)	0.10378 (4)	0.80987 (4)	0.06080 (19)	
O5	1.15357 (17)	0.53884 (17)	0.58562 (12)	0.0965 (6)	
O6	1.08908 (16)	0.18987 (14)	0.57927 (12)	0.0908 (6)	
O7	0.84584 (14)	0.49944 (11)	0.78247 (10)	0.0623 (5)	
O8	0.83861 (13)	0.74737 (10)	1.12151 (10)	0.0537 (4)	
N4	0.93679 (14)	0.37157 (11)	0.84322 (11)	0.0444 (5)	
H4	0.9932	0.3291	0.8417	0.053*	
N5	0.62508 (15)	0.71220 (13)	1.24900 (12)	0.0482 (5)	
N6	0.74652 (15)	0.86110 (13)	1.20748 (11)	0.0523 (5)	
H6	0.6911	0.8696	1.2376	0.063*	
C23	1.03238 (19)	0.48973 (17)	0.68142 (14)	0.0529 (6)	
H23	1.0154	0.5559	0.7038	0.063*	
C24	1.1014 (2)	0.4680 (2)	0.61764 (17)	0.0623 (7)	
C25	1.1221 (2)	0.3681 (2)	0.58183 (15)	0.0676 (8)	
H25	1.1677	0.3542	0.5385	0.081*	
C26	1.0758 (2)	0.2906 (2)	0.61010 (17)	0.0612 (7)	
C27	1.00969 (19)	0.31162 (17)	0.67610 (15)	0.0547 (7)	
H27	0.9795	0.2591	0.6964	0.066*	
C28	0.98907 (19)	0.40933 (17)	0.71110 (14)	0.0443 (6)	
C29	1.1378 (3)	0.6419 (2)	0.61998 (19)	0.1054 (11)	
H29A	1.0500	0.6523	0.6043	0.158*	
H29B	1.1800	0.6835	0.5924	0.158*	
H29C	1.1728	0.6599	0.6866	0.158*	
C30	1.1599 (3)	0.1633 (2)	0.51522 (19)	0.1318 (13)	
H30A	1.1195	0.1831	0.4585	0.198*	
H30B	1.1658	0.0913	0.5019	0.198*	
H30C	1.2426	0.1973	0.5417	0.198*	
C31	0.9166 (2)	0.43189 (17)	0.78087 (15)	0.0454 (6)	
C32	0.8700 (2)	0.37503 (14)	0.91011 (15)	0.0378 (5)	
C33	0.93255 (19)	0.36860 (14)	0.99944 (15)	0.0471 (6)	
H33	1.0186	0.3627	1.0155	0.057*	
C34	0.8686 (2)	0.37083 (14)	1.06550 (14)	0.0488 (6)	
H34	0.9121	0.3669	1.1257	0.059*	
C35	0.7406 (2)	0.37881 (14)	1.04267 (15)	0.0407 (6)	
C36	0.67767 (19)	0.38272 (14)	0.95225 (15)	0.0464 (6)	
H36	0.5913	0.3874	0.9357	0.056*	
C37	0.7419 (2)	0.37978 (14)	0.88621 (15)	0.0476 (6)	
H37	0.6983	0.3810	0.8253	0.057*	
C38	0.64704 (18)	0.50777 (15)	1.17087 (13)	0.0402 (6)	
C39	0.57828 (19)	0.53448 (17)	1.23420 (15)	0.0491 (6)	
H39	0.5380	0.4849	1.2520	0.059*	
C40	0.57063 (19)	0.63515 (18)	1.27021 (14)	0.0540 (6)	
H40	0.5241	0.6514	1.3127	0.065*	
C41	0.69385 (18)	0.68463 (16)	1.18968 (14)	0.0397 (6)	
C42	0.70578 (17)	0.58534 (15)	1.14923 (13)	0.0396 (5)	
H42	0.7535	0.5708	1.1074	0.047*	
C43	0.76637 (19)	0.76806 (17)	1.16986 (15)	0.0433 (6)	
C44	0.8143 (2)	0.94991 (15)	1.20025 (15)	0.0692 (8)	
H44B	0.8810	0.9292	1.1734	0.104*	
H44C	0.8488	0.9933	1.2613	0.104*	
H44A	0.7579	0.9859	1.1612	0.104*	

### Atomic displacement parameters (Å^2^)

**Table:**

	*U*^11^	*U*^22^	*U*^33^	*U*^12^	*U*^13^	*U*^23^
S1	0.0725 (5)	0.0409 (4)	0.0687 (5)	0.0063 (3)	0.0411 (4)	0.0118 (3)
O1	0.0681 (12)	0.0412 (11)	0.0543 (11)	0.0103 (8)	0.0185 (9)	0.0156 (8)
O2	0.1402 (18)	0.0447 (12)	0.0569 (12)	0.0043 (11)	0.0116 (12)	−0.0018 (10)
O3	0.0842 (14)	0.0676 (13)	0.0659 (12)	0.0430 (10)	0.0266 (10)	0.0178 (9)
O4	0.0598 (12)	0.0578 (11)	0.0464 (10)	0.0173 (9)	0.0037 (9)	0.0124 (8)
N1	0.0638 (14)	0.0518 (13)	0.0488 (13)	0.0288 (10)	0.0154 (11)	0.0152 (11)
N2	0.0547 (15)	0.0582 (15)	0.0561 (14)	0.0054 (11)	−0.0018 (12)	0.0238 (12)
N3	0.0542 (14)	0.0488 (14)	0.0595 (14)	0.0101 (11)	0.0065 (11)	0.0175 (11)
C1	0.0493 (16)	0.0411 (16)	0.0460 (15)	0.0011 (12)	0.0184 (13)	0.0056 (12)
C2	0.0459 (16)	0.0332 (16)	0.0488 (16)	0.0037 (12)	0.0204 (13)	0.0118 (13)
C3	0.0671 (18)	0.0427 (17)	0.0466 (16)	0.0053 (13)	0.0183 (14)	0.0132 (13)
C4	0.076 (2)	0.0416 (18)	0.0477 (17)	0.0048 (14)	0.0214 (15)	0.0051 (14)
C5	0.0748 (19)	0.0425 (17)	0.0576 (18)	0.0157 (14)	0.0297 (15)	0.0147 (14)
C6	0.0490 (16)	0.0458 (17)	0.0500 (16)	0.0123 (13)	0.0221 (13)	0.0145 (13)
C7	0.075 (2)	0.0595 (18)	0.0770 (19)	0.0254 (14)	0.0147 (16)	0.0302 (15)
C8	0.154 (3)	0.0420 (19)	0.077 (2)	0.0053 (18)	0.038 (2)	−0.0063 (15)
C9	0.0552 (18)	0.0508 (18)	0.0501 (17)	0.0127 (13)	0.0205 (14)	0.0123 (14)
C10	0.0500 (17)	0.0400 (15)	0.0481 (16)	0.0154 (12)	0.0119 (14)	0.0116 (12)
C11	0.0564 (19)	0.0570 (17)	0.0605 (18)	0.0165 (14)	0.0233 (15)	0.0188 (14)
C12	0.0437 (16)	0.0528 (16)	0.0585 (17)	0.0113 (12)	0.0098 (14)	0.0164 (13)
C13	0.0548 (18)	0.0324 (14)	0.0498 (16)	0.0016 (12)	0.0112 (14)	0.0096 (11)
C14	0.0611 (19)	0.0515 (16)	0.0552 (17)	0.0147 (13)	0.0237 (15)	0.0126 (13)
C15	0.0502 (17)	0.0598 (17)	0.0562 (17)	0.0208 (13)	0.0162 (15)	0.0130 (13)
C16	0.0480 (17)	0.0477 (16)	0.0448 (16)	0.0029 (13)	0.0124 (13)	0.0169 (13)
C17	0.0614 (19)	0.0573 (19)	0.0474 (16)	−0.0049 (14)	−0.0010 (13)	0.0181 (13)
C18	0.065 (2)	0.072 (2)	0.063 (2)	0.0064 (16)	−0.0072 (15)	0.0356 (17)
C19	0.0404 (16)	0.0470 (16)	0.0442 (16)	0.0057 (13)	0.0119 (13)	0.0162 (13)
C20	0.0464 (16)	0.0461 (16)	0.0399 (15)	0.0090 (12)	0.0132 (12)	0.0150 (12)
C21	0.0505 (18)	0.0479 (18)	0.0442 (16)	0.0131 (14)	0.0219 (14)	0.0151 (14)
C22	0.090 (2)	0.0500 (18)	0.075 (2)	−0.0031 (15)	0.0045 (17)	0.0058 (15)
S2	0.0754 (5)	0.0472 (4)	0.0500 (4)	0.0045 (3)	0.0053 (4)	0.0072 (3)
O5	0.1063 (16)	0.1152 (17)	0.0936 (15)	0.0018 (14)	0.0490 (13)	0.0518 (14)
O6	0.1197 (17)	0.0801 (15)	0.0861 (14)	0.0241 (12)	0.0650 (13)	0.0012 (11)
O7	0.0705 (12)	0.0642 (12)	0.0706 (12)	0.0332 (9)	0.0346 (10)	0.0331 (9)
O8	0.0566 (11)	0.0493 (10)	0.0647 (11)	0.0089 (8)	0.0343 (9)	0.0110 (8)
N4	0.0476 (13)	0.0432 (12)	0.0520 (12)	0.0203 (9)	0.0248 (11)	0.0162 (10)
N5	0.0449 (13)	0.0441 (13)	0.0593 (13)	0.0053 (10)	0.0262 (11)	0.0050 (10)
N6	0.0571 (14)	0.0381 (13)	0.0690 (14)	0.0056 (10)	0.0339 (11)	0.0070 (10)
C23	0.0537 (17)	0.0614 (18)	0.0453 (15)	0.0084 (13)	0.0118 (13)	0.0187 (13)
C24	0.0579 (18)	0.085 (2)	0.0496 (17)	−0.0002 (16)	0.0153 (14)	0.0268 (16)
C25	0.0629 (19)	0.100 (2)	0.0521 (18)	0.0106 (17)	0.0323 (15)	0.0220 (17)
C26	0.0529 (18)	0.074 (2)	0.0525 (18)	0.0119 (15)	0.0166 (14)	0.0036 (16)
C27	0.0567 (17)	0.0595 (19)	0.0496 (16)	0.0050 (13)	0.0226 (14)	0.0068 (13)
C28	0.0393 (15)	0.0528 (17)	0.0417 (15)	0.0055 (12)	0.0124 (12)	0.0117 (13)
C29	0.131 (3)	0.091 (3)	0.103 (3)	−0.014 (2)	0.028 (2)	0.049 (2)
C30	0.187 (3)	0.121 (3)	0.125 (3)	0.059 (2)	0.116 (3)	0.011 (2)
C31	0.0425 (16)	0.0466 (17)	0.0475 (16)	0.0037 (12)	0.0138 (13)	0.0107 (13)
C32	0.0397 (16)	0.0284 (13)	0.0468 (15)	0.0076 (11)	0.0155 (13)	0.0079 (11)
C33	0.0343 (15)	0.0554 (16)	0.0507 (16)	0.0089 (11)	0.0116 (13)	0.0107 (12)
C34	0.0554 (18)	0.0485 (16)	0.0423 (15)	0.0078 (13)	0.0127 (14)	0.0116 (12)
C35	0.0438 (16)	0.0297 (14)	0.0507 (16)	0.0057 (11)	0.0186 (13)	0.0070 (11)
C36	0.0343 (15)	0.0469 (16)	0.0611 (17)	0.0100 (11)	0.0167 (14)	0.0144 (12)
C37	0.0424 (16)	0.0548 (16)	0.0483 (15)	0.0105 (12)	0.0111 (13)	0.0192 (12)
C38	0.0393 (15)	0.0389 (15)	0.0434 (15)	0.0061 (11)	0.0142 (12)	0.0085 (11)
C39	0.0476 (16)	0.0501 (17)	0.0545 (16)	0.0026 (12)	0.0240 (13)	0.0105 (13)
C40	0.0509 (17)	0.0596 (18)	0.0563 (17)	0.0062 (13)	0.0317 (14)	0.0023 (14)
C41	0.0337 (14)	0.0412 (15)	0.0450 (15)	0.0061 (11)	0.0140 (12)	0.0079 (12)
C42	0.0384 (14)	0.0412 (15)	0.0413 (14)	0.0093 (11)	0.0170 (11)	0.0064 (11)
C43	0.0436 (16)	0.0383 (16)	0.0470 (16)	0.0098 (12)	0.0116 (13)	0.0091 (12)
C44	0.082 (2)	0.0438 (17)	0.090 (2)	0.0045 (14)	0.0416 (17)	0.0110 (14)

### Geometric parameters (Å, °)

**Table:**

S1—C35	1.7682 (19)	C22—H22C	0.9600
S1—C38	1.761 (2)	C22—H22A	0.9600
O1—C2	1.361 (2)	O5—C24	1.358 (2)
O1—C7	1.430 (2)	O5—C29	1.413 (3)
O2—C4	1.362 (2)	O6—C26	1.368 (3)
O2—C8	1.414 (2)	O6—C30	1.411 (2)
O3—C9	1.222 (2)	O7—C31	1.218 (2)
O4—C21	1.235 (2)	O8—C43	1.231 (2)
N1—H1	0.8600	N4—H4	0.8600
N1—C9	1.359 (2)	N4—C31	1.366 (2)
N1—C10	1.412 (2)	N4—C32	1.415 (2)
N2—C18	1.332 (3)	N5—C40	1.342 (2)
N2—C19	1.335 (2)	N5—C41	1.339 (2)
N3—H3	0.8600	N6—H6	0.8600
N3—C21	1.328 (2)	N6—C43	1.317 (2)
N3—C22	1.458 (2)	N6—C44	1.451 (2)
C1—H1A	0.9300	C23—H23	0.9300
C1—C2	1.387 (3)	C23—C24	1.387 (3)
C1—C6	1.378 (2)	C23—C28	1.396 (2)
C2—C3	1.375 (3)	C24—C25	1.394 (3)
C3—H3A	0.9300	C25—H25	0.9300
C3—C4	1.392 (3)	C25—C26	1.365 (3)
C4—C5	1.380 (3)	C26—C27	1.390 (3)
C5—H5	0.9300	C27—H27	0.9300
C5—C6	1.395 (3)	C27—C28	1.365 (2)
C6—C9	1.498 (3)	C28—C31	1.491 (3)
C7—H7B	0.9600	C29—H29A	0.9600
C7—H7C	0.9600	C29—H29B	0.9600
C7—H7A	0.9600	C29—H29C	0.9600
C8—H8C	0.9600	C30—H30A	0.9600
C8—H8B	0.9600	C30—H30B	0.9600
C8—H8A	0.9600	C30—H30C	0.9600
C10—C11	1.386 (3)	C32—C33	1.377 (2)
C10—C15	1.380 (2)	C32—C37	1.376 (2)
C11—H11	0.9300	C33—H33	0.9300
C11—C12	1.371 (3)	C33—C34	1.383 (2)
C12—H12	0.9300	C34—H34	0.9300
C12—C13	1.391 (2)	C34—C35	1.381 (2)
C13—C14	1.378 (3)	C35—C36	1.383 (3)
C13—S2	1.768 (2)	C36—H36	0.9300
C14—H14	0.9300	C36—C37	1.384 (2)
C14—C15	1.383 (3)	C37—H37	0.9300
C15—H15	0.9300	C38—C39	1.385 (2)
C16—C17	1.382 (3)	C38—C42	1.376 (2)
C16—C20	1.381 (3)	C39—H39	0.9300
C16—S2	1.757 (2)	C39—C40	1.369 (2)
C17—H17	0.9300	C40—H40	0.9300
C17—C18	1.380 (3)	C41—C42	1.378 (2)
C18—H18	0.9300	C41—C43	1.509 (3)
C19—C20	1.388 (2)	C42—H42	0.9300
C19—C21	1.507 (3)	C44—H44B	0.9600
C20—H20	0.9300	C44—H44C	0.9600
C22—H22B	0.9600	C44—H44A	0.9600
			
O1—C2—C1	115.0 (2)	O5—C24—C23	124.4 (3)
O1—C2—C3	124.5 (2)	O5—C24—C25	115.1 (2)
O1—C7—H7B	109.5	O5—C29—H29A	109.5
O1—C7—H7C	109.5	O5—C29—H29B	109.5
O1—C7—H7A	109.5	O5—C29—H29C	109.5
O2—C4—C3	114.4 (2)	O6—C26—C27	114.7 (2)
O2—C4—C5	124.6 (2)	O6—C30—H30A	109.5
O2—C8—H8C	109.5	O6—C30—H30B	109.5
O2—C8—H8B	109.5	O6—C30—H30C	109.5
O2—C8—H8A	109.5	O7—C31—N4	122.79 (19)
O3—C9—N1	123.0 (2)	O7—C31—C28	122.51 (19)
O3—C9—C6	122.3 (2)	O8—C43—N6	124.05 (19)
O4—C21—N3	123.5 (2)	O8—C43—C41	120.4 (2)
O4—C21—C19	121.2 (2)	N4—C31—C28	114.70 (19)
N1—C9—C6	114.6 (2)	N5—C40—C39	125.08 (19)
N2—C18—C17	124.9 (2)	N5—C40—H40	117.5
N2—C18—H18	117.5	N5—C41—C42	123.90 (18)
N2—C19—C20	124.2 (2)	N5—C41—C43	117.27 (19)
N2—C19—C21	117.5 (2)	N6—C43—C41	115.56 (19)
N3—C21—C19	115.3 (2)	N6—C44—H44B	109.5
N3—C22—H22B	109.5	N6—C44—H44C	109.5
N3—C22—H22C	109.5	N6—C44—H44A	109.5
N3—C22—H22A	109.5	C23—C24—C25	120.5 (2)
C1—C6—C5	120.9 (2)	C23—C28—C31	118.6 (2)
C1—C6—C9	121.8 (2)	C24—O5—C29	118.1 (2)
C2—O1—C7	116.82 (17)	C24—C23—H23	121.0
C2—C1—H1A	120.2	C24—C23—C28	118.1 (2)
C2—C3—H3A	120.3	C24—C25—H25	119.9
C2—C3—C4	119.4 (2)	C25—C26—O6	125.5 (2)
C3—C2—C1	120.5 (2)	C25—C26—C27	119.8 (2)
C4—O2—C8	118.42 (19)	C26—O6—C30	117.80 (19)
C4—C3—H3A	120.3	C26—C25—C24	120.3 (2)
C4—C5—H5	120.7	C26—C25—H25	119.9
C4—C5—C6	118.6 (2)	C26—C27—H27	120.0
C5—C4—C3	121.0 (2)	C27—C28—C23	121.31 (19)
C5—C6—C9	117.3 (2)	C27—C28—C31	120.11 (19)
C6—C1—H1A	120.2	C28—C23—H23	121.0
C6—C1—C2	119.6 (2)	C28—C27—C26	120.0 (2)
C6—C5—H5	120.7	C28—C27—H27	120.0
H7B—C7—H7C	109.5	H29A—C29—H29B	109.5
H7B—C7—H7A	109.5	H29A—C29—H29C	109.5
H7C—C7—H7A	109.5	H29B—C29—H29C	109.5
H8C—C8—H8B	109.5	H30A—C30—H30B	109.5
H8C—C8—H8A	109.5	H30A—C30—H30C	109.5
H8B—C8—H8A	109.5	H30B—C30—H30C	109.5
C9—N1—H1	117.5	C31—N4—H4	118.2
C9—N1—C10	125.04 (19)	C31—N4—C32	123.61 (17)
C10—N1—H1	117.5	C32—N4—H4	118.2
C10—C11—H11	119.9	C32—C33—H33	119.7
C10—C15—C14	120.7 (2)	C32—C33—C34	120.7 (2)
C10—C15—H15	119.7	C32—C37—C36	120.4 (2)
C11—C10—N1	121.4 (2)	C32—C37—H37	119.8
C11—C12—H12	119.5	C33—C32—N4	119.8 (2)
C11—C12—C13	120.9 (2)	C33—C34—H34	119.8
C12—C11—C10	120.2 (2)	C34—C33—H33	119.7
C12—C11—H11	119.9	C34—C35—S1	121.10 (18)
C12—C13—S2	119.56 (18)	C34—C35—C36	118.69 (19)
C13—C12—H12	119.5	C35—C34—C33	120.5 (2)
C13—C14—H14	119.8	C35—C34—H34	119.8
C13—C14—C15	120.4 (2)	C35—C36—H36	119.7
C14—C13—C12	118.7 (2)	C35—C36—C37	120.6 (2)
C14—C13—S2	121.66 (18)	C36—C35—S1	120.15 (17)
C14—C15—H15	119.7	C36—C37—H37	119.8
C15—C10—N1	119.5 (2)	C37—C32—N4	121.0 (2)
C15—C10—C11	119.0 (2)	C37—C32—C33	119.08 (19)
C15—C14—H14	119.8	C37—C36—H36	119.7
C16—C17—H17	120.6	C38—S1—C35	103.42 (9)
C16—C20—C19	119.1 (2)	C38—C39—H39	120.6
C16—C20—H20	120.5	C38—C42—C41	119.88 (17)
C16—S2—C13	102.47 (10)	C38—C42—H42	120.1
C17—C16—S2	118.17 (19)	C39—C38—S1	118.50 (15)
C17—C18—H18	117.5	C39—C40—H40	117.5
C18—N2—C19	115.4 (2)	C40—C39—C38	118.88 (18)
C18—C17—C16	118.8 (2)	C40—C39—H39	120.6
C18—C17—H17	120.6	C41—N5—C40	115.00 (18)
C19—C20—H20	120.5	C41—C42—H42	120.1
C20—C16—C17	117.5 (2)	C42—C38—S1	124.27 (15)
C20—C16—S2	124.28 (18)	C42—C38—C39	117.22 (19)
C20—C19—C21	118.4 (2)	C42—C41—C43	118.77 (18)
C21—N3—H3	118.9	C43—N6—H6	118.7
C21—N3—C22	122.1 (2)	C43—N6—C44	122.66 (17)
C22—N3—H3	118.9	C44—N6—H6	118.7
H22B—C22—H22C	109.5	H44B—C44—H44C	109.5
H22B—C22—H22A	109.5	H44B—C44—H44A	109.5
H22C—C22—H22A	109.5	H44C—C44—H44A	109.5
			
S1—C35—C36—C37	−177.77 (14)	S2—C13—C14—C15	178.39 (16)
S1—C38—C39—C40	179.83 (17)	S2—C16—C17—C18	178.32 (16)
S1—C38—C42—C41	−179.13 (15)	S2—C16—C20—C19	−177.09 (14)
O1—C2—C3—C4	179.82 (17)	O5—C24—C25—C26	179.0 (2)
O2—C4—C5—C6	−178.31 (19)	O6—C26—C27—C28	−179.1 (2)
N1—C10—C11—C12	179.40 (19)	N4—C32—C33—C34	−179.35 (17)
N1—C10—C15—C14	−179.2 (2)	N4—C32—C37—C36	179.74 (17)
N2—C19—C20—C16	−2.2 (3)	N5—C41—C42—C38	−1.4 (3)
N2—C19—C21—O4	−177.59 (19)	N5—C41—C43—O8	174.55 (19)
N2—C19—C21—N3	3.5 (3)	N5—C41—C43—N6	−5.3 (3)
C1—C2—C3—C4	−1.8 (3)	C23—C24—C25—C26	−0.8 (4)
C1—C6—C9—O3	−149.3 (2)	C23—C28—C31—O7	34.5 (3)
C1—C6—C9—N1	32.3 (3)	C23—C28—C31—N4	−144.92 (19)
C2—C1—C6—C5	2.1 (3)	C24—C23—C28—C27	−2.4 (3)
C2—C1—C6—C9	−176.02 (18)	C24—C23—C28—C31	179.0 (2)
C2—C3—C4—O2	−179.76 (18)	C24—C25—C26—O6	179.3 (2)
C2—C3—C4—C5	2.2 (3)	C24—C25—C26—C27	−1.1 (4)
C3—C4—C5—C6	−0.5 (3)	C25—C26—C27—C28	1.3 (3)
C4—C5—C6—C1	−1.7 (3)	C26—C27—C28—C23	0.5 (3)
C4—C5—C6—C9	176.49 (19)	C26—C27—C28—C31	179.1 (2)
C5—C6—C9—O3	32.5 (3)	C27—C28—C31—O7	−144.2 (2)
C5—C6—C9—N1	−145.87 (19)	C27—C28—C31—N4	36.4 (3)
C6—C1—C2—O1	178.23 (15)	C28—C23—C24—O5	−177.3 (2)
C6—C1—C2—C3	−0.3 (3)	C28—C23—C24—C25	2.5 (3)
C7—O1—C2—C1	−177.90 (17)	C29—O5—C24—C23	0.6 (4)
C7—O1—C2—C3	0.6 (3)	C29—O5—C24—C25	−179.1 (2)
C8—O2—C4—C3	177.17 (18)	C30—O6—C26—C25	2.2 (4)
C8—O2—C4—C5	−4.8 (3)	C30—O6—C26—C27	−177.3 (2)
C9—N1—C10—C11	36.4 (3)	C31—N4—C32—C33	−139.0 (2)
C9—N1—C10—C15	−145.8 (2)	C31—N4—C32—C37	44.2 (3)
C10—N1—C9—O3	4.0 (4)	C32—N4—C31—O7	5.9 (3)
C10—N1—C9—C6	−177.61 (19)	C32—N4—C31—C28	−174.73 (18)
C10—C11—C12—C13	−0.7 (3)	C32—C33—C34—C35	0.5 (3)
C11—C10—C15—C14	−1.3 (3)	C33—C32—C37—C36	2.9 (3)
C11—C12—C13—C14	−0.6 (3)	C33—C34—C35—S1	178.18 (15)
C11—C12—C13—S2	−178.15 (16)	C33—C34—C35—C36	1.1 (3)
C12—C13—C14—C15	0.9 (3)	C34—C35—C36—C37	−0.7 (3)
C12—C13—S2—C16	−68.06 (18)	C35—S1—C38—C39	175.66 (17)
C13—C14—C15—C10	0.1 (3)	C35—S1—C38—C42	−5.7 (2)
C14—C13—S2—C16	114.47 (18)	C35—C36—C37—C32	−1.4 (3)
C15—C10—C11—C12	1.6 (3)	C37—C32—C33—C34	−2.5 (3)
C16—C17—C18—N2	−0.3 (4)	C38—S1—C35—C34	96.53 (17)
C17—C16—C20—C19	1.8 (3)	C38—S1—C35—C36	−86.45 (18)
C17—C16—S2—C13	161.70 (16)	C38—C39—C40—N5	0.1 (3)
C18—N2—C19—C20	1.3 (3)	C39—C38—C42—C41	−0.5 (3)
C18—N2—C19—C21	−177.12 (18)	C40—N5—C41—C42	2.5 (3)
C19—N2—C18—C17	0.0 (3)	C40—N5—C41—C43	−174.82 (18)
C20—C16—C17—C18	−0.6 (3)	C41—N5—C40—C39	−1.8 (3)
C20—C16—S2—C13	−19.4 (2)	C42—C38—C39—C40	1.1 (3)
C20—C19—C21—O4	3.9 (3)	C42—C41—C43—O8	−2.9 (3)
C20—C19—C21—N3	−174.95 (17)	C42—C41—C43—N6	177.22 (19)
C21—C19—C20—C16	176.16 (17)	C43—C41—C42—C38	175.86 (17)
C22—N3—C21—O4	−3.2 (3)	C44—N6—C43—O8	−3.8 (3)
C22—N3—C21—C19	175.66 (17)	C44—N6—C43—C41	176.07 (17)

### Hydrogen-bond geometry (Å, °)

**Table:**

*D*—H···*A*	*D*—H	H···*A*	*D*···*A*	*D*—H···*A*
N1—H1···O4^i^	0.86	2.12	2.941 (2)	159
N3—H3···O7^ii^	0.86	2.22	2.922 (2)	138
N4—H4···O8^iii^	0.86	2.15	2.985 (2)	163
N6—H6···O3^iv^	0.86	2.30	2.957 (2)	133

Symmetry codes: (i) −*x*+1, −*y*, −*z*+1; (ii) *x*, *y*−1, *z*; (iii) −*x*+2, −*y*+1, −*z*+2; (iv) *x*, *y*+1, *z*+1.
